# The susceptibility of *FSHB* -211G > T and *FSHR* G-29A, 919A > G, 2039A > G polymorphisms to men infertility: an association study and meta-analysis

**DOI:** 10.1186/s12881-017-0441-4

**Published:** 2017-08-01

**Authors:** Qiuyue Wu, Jing Zhang, Peiran Zhu, Weijun Jiang, Shuaimei Liu, Mengxia Ni, Mingchao Zhang, Weiwei Li, Qing Zhou, Yingxia Cui, Xinyi Xia

**Affiliations:** Institute of Laboratory Medicine, Jinling Hospital, Nanjing University School of Medicine, Nanjing, 210002 People’s Republic of China

**Keywords:** Male infertility, *FSHB* gene, *FSHR* gene, Polymorphisms, Case-control study, Meta-analysis

## Abstract

**Background:**

Male infertility is a complex disorder caused by genetic, developmental, endocrine, or environmental factors as well as unknown etiology. Polymorphisms in the follicle stimulating hormone beta subunit (*FSHB)* (rs10835638, c.-211G > T) and follicle stimulating hormone receptor *(FSHR)* (rs1394205, c.-29G > A; rs6165, c.919A > G; rs6166, c.2039 A > G) genes might disturb normal spermatogenesis and affect male reproductive ability.

**Methods:**

To further ascertain the aforementioned effects, we conducted a case-control study of 255 infertile men and 340 fertile controls from South China using the Mass ARRAY method, which was analyzed by the t-tests and logistic regression analysis using SPSS for Windows 14.0. In addition, a meta-analysis was performed by combining our results with previous reports using STATA 12.0.

**Results:**

In the *FSHB* or *FSHR* gene single nucleotide polymorphism (SNP) evaluation, no statistically-significant difference was found in the frequency of allelic variants or in genotype distribution between cases and controls. However, a significant association for the comparison of GAA (*P*: 0.022, OR: 0.63, 95%CI: 0.43–0.94) was seen between the oligozoospermia and controls in haplotype analysis of rs1394205/rs6165/rs6166. In the meta-analysis, rs6165G allele and rs6166 GG genotype were associated with increased risk of the male infertility.

**Conclusions:**

This study suggested that *FSHR* GAA haplotype would exert protective effects against male sterility, which indicated that the combination of three SNP genotypes of *FSHR* was predicted to have a much stronger impact than either one alone. Then in the meta-analysis, a significant association was seen between *FSHR* rs6165, rs6166 polymorphisms and male infertility. In terms of male infertility with multifactorial etiology, further studies with larger sample sizes and different ethnic backgrounds or other risk factors are warranted to clarify the potential role of *FSHB* and *FSHR* polymorphisms in the pathogenesis of male infertility.

## Background

Worldwide, approximately 15% of couples cannot have a child, and in half of the cases the reason is the result of male infertility [1], which is a complex disorder caused by genetic, developmental, endocrine, or environmental factors or still unknown etiology [2]. It has been demonstrated that approximately 30% of infertility cases could be attributed to genetic defects, such as Klinefelter syndrome (KS), disorders of sexual development (DSD), or congenital absence of the vas deferens. Therefore, it is necessary to prevent and treat male infertility from the genetic viewpoint [3].

Nowadays, increasing numbers of researchers have focused their attention on the relationship between genetic polymorphisms and male infertility, including methylene-tetrahydrofolate reductase (*MTHFR*) [4, 5], deleted in azoospermia like gene (*DAZL*) [6, 7], androgen receptor gene (*A*R) [8], glutathione S-transferase genes (*GSTM1, GSTT1, GSTP1*) [9–11] and follicle stimulating hormone receptor gene (*FSHR*, OMIM:136,435) [12–14], or FSH beta subunit (*FSHB*,OMIM:136,530) [15].

FSH acts as a spermatogonial survival factor in the adult testis and is also a necessary signal for Sertoli cell proliferation, a process that occurs during fetal, neonatal, and prepubertal life [16]. FSH is a double-stranded glycoprotein consisting of two chains, including 92 amino acids forming the α chain and 111 amino acids comprising the β chain, which are coupled by a non-covalent bond [17]. Most clinical studies reported so far only focused on the single nucleotide polymorphism (SNP) rs10835638 (c.-211G > T), which is located in an element of the *FSHB* promoter influencing gene transcription by affecting the binding of the LHX3 homeodomain transcription factor [15, 18, 19]. In order to exert its stimulatory effect, FSH binds to its receptor, the FSHR expressed on the membrane of granulosa cells (GCs) in the ovary and Sertoli cells in the testis to bring about folliculogenesis [20] and spermatogenesis [21], respectively. The *FSHR* gene is located on chromosome 2p21 and consists of 9 introns and 10 exons [22]. Given the significant role of FSH in fertility, genetic abnormalities of the *FSHR* could cause infertility. *FSHR* SNP rs6166 exchanges asparagine (Asn) for serine (Ser) in the intracellular domain of the receptor, introducing a potential phosphorylationsite; while rs6165 replaces threonine (Thr) with alanine (Ala), resulting in a change from a polar (T) to a nonpolar, hydrophobic (A) amino acid and removing a potential O-linked glycosylation site [22]. A previous studies in human granulosa-lutein cells (hGLC) has revealed that the two *FSHR* polymorphisms, blunted ERK1/2 (extracellular regulated protein kinases 1/2) activation, which indicated that Ala307-Ser680 *FSHR* was indeed less “active” in vitro, providing a molecular explanation for the clinical data [23]. Clinical studies suggested that those men with both mutated *FSHR* variants may have significantly higher basal serum FSH levels. SNP (rs1394205) exchanges a nucleotide in the promoter region of the *FSHR* gene (c.-29G > A), resulting in a significant 56% decrease in the transcriptional promoter activity of A allele [18]. In summary, SNPs involved either in signal transduction (*FSHR* exon 10) or in transcriptional activity (*FSHR* and *FSHB* promoter) in vitro could result in an overall change of FSH action.

Among these candidates, the linked SNPs at positions 307 and 680 in exon 10 of the *FSHR* gene have been extensively investigated as the potential cause of male infertility in various ethnic populations [14, 22, 24, 25], and the results largely failed to demonstrate significant associations. Polymorphism rs1394205 is located in the 5′-untranslated region (5′-UTR) of *FSHR* (c.-29G/A), which has been reported to affect the serum level of FSH [22]. For the *FSHB,* rs10835638 is a -211G > T polymorphism located in the element of the *FSHB* promoter, which has been reported to be associated with lower testicular volume, lower sperm count, lower testosterone, and higher luteinizing hormone (LH) serum levels [18, 22]. However, the associations between these SNPs in the *FSHR* gene and *FSHB* and male infertility remain uncertain.

To further verify the effects of the polymorphisms (*FSHB* rs10835638, *FSHR* rs6165, rs6166, rs1394205) on the risk of male infertility and to quantify potential heterogeneity between various studies, we conducted a case-control study of 255 infertile men and 340 healthy controls from South China, as well as performing a meta-analysis of the results of previous reports and this study.

## Methods

### Study population

Only men of Han-Chinese ethnicity were recruited between April 2013 and July 2015 among the participants in the Institute of Laboratory Medicine, Jinling Hospital, Nanjing University School of Medicine. This study population was consisted of 340 fertile men as healthy control, who had at least 1 child in the last year by direct survey and lacked any history of requiring assisted reproduction technology, and 255 infertile men, including 166 with azoospermia or severe oligozoospermia (sperm concentration < 5 ×106/ml), and 89 with oligozoospermia (sperm concentration5–15×106/ml), with at least 1 year of infertility. Individuals with known causes of infertility including genetic factors (chromosome anomalies), AZF microdeletions, clinical factors (varicocele, crytorchidism), obstructive azoospermia and infections were excluded from this study. All controls and cases were ethnic Han-Chinese.

### Evaluations

All the enrolled patients were examined at least two semen analyses according to the World Health Organization guidelines (WHO, 2010). In brief, after ejaculation, the semen was incubated at 37 °C for 30–40 min for liquefaction. Semen volume was estimated by weighing the collection tube with the semen sample and subsequently subtracting the predetermined weight of the empty tube assuming 1 g = 1 mL. For assessment of the sperm concentration, the samples were diluted in a solution of 0.6 mol/L NaHCO_3_ and 0.4% (*v*/v) formaldehyde in distilled water. The sperm concentration was assessed using the improved Neubauer haemocytometers.

In addition, the participants underwent medical and andrological examination including medical history, hormonal analysis for the measurement of serum LH, FSH, total testosterone (T), Estradiol (E_2_), Prolactin, karyotype, and Y chromosome microdeletion screening. Karyotype and Y chromosome microdeletion were determined by G-banding in lymphocytes and multiplex polymerase chain reaction (PCR) using primers (sY84, sY86, sY127, sY134, sY254, sY255, SRY, ZFX/ZFY) specific for the diagnosis of microdeletion of the AZFa, AZFb, and AZFc regions, respectively. Then serum hormone levels were detected by chemiluminescent microparticle immunoassay on an Abott-ARCHITECT Immunoanalyser (Abbott Laboratories Abbott Park, IL, USA). The intra- and interassay coefficients of variation (CV) for measurement of both FSH and LH were 3 and 4.5%, for total testosterone <8% and <5%, for estradiol 7.5% and 13%, respectively.

### Genomic DNA extraction and genotyping

Genomic DNA was extracted from the peripheral blood of the 255 infertile men and 340 fertile men using a blood DNA extraction kit (TIANGEN, Beijing, China). The DNA purity was measured by spectrometry (DU530UV/VIS spectrophotometer, Beckman Instruments, Fullerton, CA, USA). Genotyping was performed using the Mass ARRAY platform [26, 27]. In brief, SNPs were detected by Sequenom Mass ARRAY RS1000 according to the standard protocol. Multiplexed SNP Mass EXTENDED assay was designed by Sequenom Mass ARRAY Assay Design 3.0 Software version. The primers were: for codon 307 polymorphism, forward primers 5′-TTCTACCCTGCACAAAGACAG-3′, reverse primer 5′- AATCCTCTGCTGTAGCTGGAC-3′; for codon 680 polymorphism, forward primers 5′-CACTGTCCACAACACCCATCC-3′, reverse primer 5′- ACCCTTCAAAGGCAAGACTGA-3′; for nucleotide position −29 polymorphism, forward primer 5′-ACGTTGGATGCAGGGCCATAATTATGCATC-3′, reverse primer 5′-ACGTTGGATGTGTGGAGCTTCTGAGATCTG-3′; for nucleotide position −211 polymorphism, forward primer 5′-ACGTTGGATGCTAAAGTAGTCTAAACGCAG-3′, reverse primer 5′-ACGTTGGATGAGTGGGTGTGCTACTGTATC-3′. Finally, data management and analyses was performed by Sequenom Mass ARRAY Analyzer 4.0 system. Then the effectiveness of this method was verified by direct sequencing analysis (ABI PRISM3730XL DNA Sequencer; Applied Biosystems) of the first 100 DNA samples.

### Meta-analysis

#### Study selection

To identify the related articles, a comprehensive systematic searching was performed in the PubMed, Web of Science and the Chinese National Knowledge Infrastructure (CNKI) database, using the search words “*FSHR* rs6165”, “*FSHR* rs6166”, “*FSHR* rs1394205”, “polymorphism” and “male infertility”. Included studies had to meet the following criteria: (1) evaluation of the *FSHR* rs1394205, rs6165, rs6166 and male infertility; (2) involving in human beings; (3) a case-control study; (4) with detailed genotype frequency of cases and controls or obtained the article text.

### Data extraction and verification

Two authors (Qiuyue Wu and Jing Zhang) extracted the data independently that met the inclusion criteria and reached the consensus for any controversy. The main characteristics of enrolled studies were listed, including: (I) First author’s name, (II) Year of publication, (III) Race, (IV) Control sources, (V) Genotyping methods, (VI) Polymorphism sites, (VII) Control/Case counts,(VII) Genotype counts (control/case), (IX) Hard-Weinberg equilibrium (HWE) in the controls and (X) Clinical diagnose of cases.

### Statistical analysis and meta-analysis

#### Analysis of genetic data

T-test was used to measure the differences in the distributions of clinical characteristics, including age, hormone and sperm parameters between groups and cases. The difference of *FSHR* rs1394205, rs6165 and rs6166 polymorphism and *FSHB* rs10835638 polymorphism between the infertile and fertile groups was calculated using a logistic regression model, SNPs coded as three categories: wild-type homozygote (WW, reference), the heterozygous (WR) and rare allele homozygote (RR), which yielded a *p* value and odds ratio (OR) with the corresponding 95% confidence interval (CI), using SPSS for Windows 14.0 (SPSS, Inc., Chicago, Illinois). And *P* < 0.05 was considered statistically significant. The linkage disequilibria and haplotypes were analyzed with SHEsis software (http://analysis.bio-x.cn/SHEsisMain.htm). Because only the data of Age was collected completely, genetic association tests have been adjusted for Age effects.

#### Statistics for meta-analysis

This study and other related case-control studies were combined for the meta-analysis, which was performed using STATA 12.0 (STATA Corporation LP, College Station, TX, USA). Odds ratio (OR) and 95% confidence interval (CI) were calculated to estimate the associations of *FSHR* rs6165、rs6166、rs1394205 polymorphisms with male infertility susceptibility based on three genetic models, including co-dominant model [the rare allele homozygote (RR) vs. wild-type homozygote (WW), the heterozygous (WR) vs. WW], dominant model (RR + WR vs. WW), recessive model (RR vs. WW + WR). In addition, stratified analyses were performed by HWE (>0.05 and <0.05), race (Caucasian, Asian and Brazilian population) and case counts (>200 and <200). HWE < 0.05 indicates unbalanced distribution in the frequency of the population. So studies with SNPs with HWE < 0.05 were removed from meta-analysis. Heterogeneity across the studies was evaluated by t-test test based on Q test and was considered significant if *P*-value for heterogeneity (*P*_h_) was <0.05. A fixed-effect model with no heterogeneity (*P*_h_ > 0.05 or I_2_ < 50%) using the Mantel–Haenszel method and a random effects model with a high heterogeneity (*P*_h_ < 0.05 or I_2_ > 50%) using the DerSimonian and Laird method were used to pool the results. Moreover, a sensitivity analysis, by which a single study in the meta-analysis was deleted each time to determine the influence of the individual data set to the overall pooled OR, was performed to assess the stability of the results. To test the publication bias, Begg’s Funnel plots and Egger’s linear regression test were applied. HWE in the controls of each study was calculated using a web-based program (http://ihg.gsf.de/cgi-bin/hw/hwa1.pl).

## Results

### Clinical characteristics of the study population

A total of 255 infertile men and 340 infertile men were collected in this case-control study. The clinical charactereristics of the participants were presented in Table 1. The observed frequencies of all tested genotypes in controls were in agreement with the HWE (*P*: 0.538 for *FSHB* rs10835638, *P*: 0.975 for *FSHR* rs1394205, *P*: 0.884 for rs6165, *P*: 0.391 for rs6166, respectively). The serum FSH and LH concentrations in fertile men were 4.72 ± 2.51 IU/L and 3.37 ± 1.46 IU/L, whereas the FSH and LH values in infertile patients were 15.73 ± 16.15 IU/L and 6.46 ± 4.98 IU/L, respectively. The FSH and LH levels in the infertile patients were significantly higher than that in the fertile men (*P* < 0.05). And fertile men had higher sperm concentration and sperm motility compared with the infertile men. No statistical difference was observed in the other indicators.

**Table 1:** General characteristics of the study group

Clinical parameters	Fertile controls (*n* = 340)		Infertile cases (*n* = 255)		Azoospermic or severe oligozoospermia (*n* = 166)		Oligospermia (*n* = 89)	
	number	Mean ± SD	number	Mean ± SD	number	Mean ± SD	number	Mean ± SD
Age (years)	340	28.37 ± 4.23	255	28.49 ± 4.51	166	28.45 ± 4.34	89	28.56 ± 4.83
T (9.4-37 nmol/L)	32	12.43 ± 4.83	71	12.34 ± 4.74	52	13.18 ± 4.60	19	13.78 ± 5.20
FSH (1–5.5 IU/L)	32	4.72 ± 2.51	70	**15.73 ± 16.15**	52	**19.28 ± 17.32**	18	5.46 ± 2.80
LH (1–6.3 IU/L)	32	3.37 ± 1.46	70	**6.46 ± 4.98**	52	**7.42 ± 5.41**	18	3.69 ± 1.49
E_2_ (58.6–194.2 pmol/L)	24	103.87 ± 77.35	42	112.00 ± 68.07	29	107.10 ± 71.75	13	122.92 ± 60.25
pH (7.2–7.4)	177	7.38 ± 0.06	89	7.37 ± 0.07	40	7.38 ± 0.53	49	7.36 ± 0.78
Semen volume (1.5-6 ml)	177	3.51 ± 1.39	89	3.78 ± 1.77	40	3.52 ± 1.27	49	3.98 ± 2.09
Sperm concentration (≥15 × 10^6^/ml)	340	72.77 ± 45.21	255	**3.15 ± 4.52**	166	**0.27 ± 0.88**	89	**8.53 ± 3.55**
Sperm motility (PR ≥ 32%)	340	42.02 ± 9.04	255	**11.76 ± 17.76**	166	**1.32 ± 4.92**	89	**31.24 ± 16.61**

*SD* standard deviation. Bold numbers was considered to be statistically significant compared with the controls

### Case-control study of *FSH* and *FSHR* gene polymorphisms

Logistic regression analysis revealed that, when the *FSHR* SNPs at nucleotide −211, −29, codon 307 and codon 680 was separately analyzed, no statistically significant difference was found in the frequency of allelic variants or in genotype distribution between cases and controls, as showed in Table 2.

**Table 2:** Allele and genotype frequencies of the *FSHR* rs6165, rs6166 and rs1394205 genotypes and *FSHB* rs10835638 genotypes in the infertile and fertile groups

Genotype	Control (*n* = 340) frequency	Case (*n* = 255)			Sperm concentration(<5 ×10^6^/ml) (*n* = 166)			Sperm concentration(5–15 ×10^6^/ml) (*n* = 89)		
		frequency	*P*	OR(95%CI)	frequency	*P*	OR(95%CI)	frequency	*P*	OR(95%CI)
*FSHR* rs6165Thr307Ala										
AA	0.479	0.416	0.292	ref	0.422	0.452	ref	0.404	0.448	ref
AG	0.424	0.471	0.154	1.28(0.91–1.81)	0.464	0.269	1.25(0.84–1.85)	0.483	0.232	1.35 (0.82–2.22)
GG	0.097	0.113	0.285	1.35 (0.77–2.36)	0.114	0.355	1.35 (0.72–2.53)	0.113	0.429	1.38 (0.62–3.05)
*FSHR* rs6166 Asn680Ser										
AA	0.497	0.475	0.466	ref	0.476	0.418	ref	0.472	0.836	ref
AG	0.429	0.424	0.842	1.04 (0.74–1.46)	0.416	0.946	1.01 (0.68–1.50)	0.438	0.770	1.08 (0.66–1.75)
GG	0.074	0.101	0.219	1.45 (0.80–2.64)	0.108	0.199	1.54 (0.80–3.00)	0.100	0.561	1.29 (0.54–3.07)
*FSHR* rs1394205 -29G > A										
GG	0.229	0.208	0.704	ref	0.241	0.328	ref	0.146	0.136	ref
GA	0.500	0.494	0.675	1.09 (0.72–1.66)	0.434	0.427	0.83 (0.52–1.32)	0.607	0.055	1.91 (0.99–3.71)
AA	0.271	0.298	0.408	1.22 (0.76–1.93)	0.325	0.603	1.14 (0.69–1.90)	0.247	0.348	1.43 (0.68–3.04)
*FSHB* rs10835638 -211G > T										
GG	0.935	0.929	0.993	ref	0.928	0.996	ref	0.933	-	ref
GT	0.065	0.067	0.908	1.04 (0.54–2.00)	0.066	0.930	1.03 (0.49–2.19)	0.067	0.935	1.04 (0.41–2.65)

*OR* odds ratio, *CI* confidence interval; Ref: control by heterozygous genotypes and rare homozygous

Haplotype analysis demonstrated that there was a strong linkage disequilibrium (LD) between SNPs 307/680 (D′ = 0.983, r2 = 0.852), weak LD between SNPs −29/307 (D’ = 0.138, r2 = 0.01), and SNPs −29/680 (D’ = 0.195, r2 = 0.018) locuses. A significant association for the comparison of GAA (*P*: 0.022, OR: 0.63, 95%CI: 0.43–0.94) was seen between the oligozoospermia and controls in haplotype analysis of rs1394205/rs6165/rs6166 (Table 3). Furthermore, we evaluated the effects of *FSHB*-*FSHR* interactions on the risk of infertility, no significant association was observed in the case and control group.

**Table 3:** Haplotype analysis of the *FSHR* three SNPs rs6165, rs6166 and rs1394205

Haplotype	Control (*n* = 340) Frequency	Case (*n* = 255)			Sperm concentration(<5 ×10^6^/ml) (*n* = 166)			Sperm concentration(5–15 ×10^6^/ml) (*n* = 89)		
		Frequency	*P*	OR(95%CI)	Frequency	*P*	OR(95%CI)	Frequency	*P*	OR(95%CI)
*FSHR* rs1394205/rs6165/rs6166										
AAA	0.376	0.382	0.794	1.03 (0.81–1.31)	0.366	0.652	0.94 (0.72–1.23)	0.427	0.163	1.27 (0.91–1.79)
AGG	0.121	0.133	0.539	1.11 (0.79–1.57)	0.146	0.308	1.22 (0.83–1.79)	0.100	0.467	0.82 (0.48–1.41)
GAA	0.309	0.269	0.133	0.82 (0.64–1.06)	0.287	0.408	0.89 (0.66–1.18)	0.219	**0.022**	**0.63 (0.43–0.94)**
GGG	0.161	0.181	0.351	1.16 (0.85–1.57)	0.170	0.764	1.06 (0.74–1.50)	0.209	0.111	1.40 (0.92–2.13)
*FSHR* rs6165/rs6166										
AA	0.685	0.669	0.430	0.92 (0.74–1.14)	0.653	0.256	0.85 (0.64–1.13)	0.678	0.729	0.96 (0.76–1.22)
GG	0.282	0.296	0.579	1.06 (0.86–1.32)	0.317	0.256	1.18 (0.89–1.57)	0.285	0.946	1.01 (0.79–1.29)

Bold numbers mean statistically significant results

### Meta-analysis of *FSHR* gene polymorphisms in infertile men

**Fig. 1:**
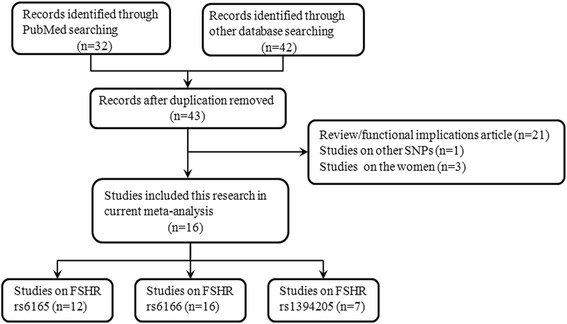
Flow chart of studies identified with inclusion and exclusion criteria

A total of 15 studies, 4 studies from Asian population [24, 28–30] and 11 studies from non-Asian population [1, 13, 14, 16, 17, 22, 31–35], met the inclusion criteria (Fig. 1). Including our study, the main characteristics of 12 case-control studies (2903 controls and 2564 cases) on the rs6165, 16 studies (4320 controls and 3728 cases) on the rs6166, and 7 studies on the rs1394205 (2776 controls and 2048 cases) were showed in Table 4. To determine the SNPs, three different genotyping methods were applied, including TaqMan assays, sequencing and PCR-RFLP. In addition, the sources of controls of these studies were mainly hospital population. The distribution of genotypes in the controls of HWE by the Gharesi-Fard et al. 2015 [13], Pengo et al., 2006 [22], Balkan et al., 2010 [17] and Ghirelli-Filho et al., 2012 [1] reported, were <0.05, which were divided into subgroup in this meta-analysis.

**Table 4:** Main characteristics of all studies on the genotype of *FSHR* rs6165, rs6166 and rs1394205 included in the meta-analysis

Author	Year	Race	Control source	Genotyping methods	Polymorphism sites	Control/ Case counts	Genotype counts (control/case)	HWE (Control)	Clinical diagnosis of cases
Gharesi-Fard B	2015	Caucasian	HP	PCR-RELP	rs6165 rs6166	200/212	AA:47/64, AG:85/91, GG:68/57; AA:40/33,AG:107/128,GG:53/51;	0.047 0.291	This case control study was performed on 212 primary azoospermic patients and 200 healthy men. Azoospermia was confirmed based on two separate semen analysis. Inclusion criteria for NOA were, having no history of genital infections and existence of bilateral vas deferens and the exclusion criteria were, having history of surgery or vasectomy. All OA cases were selected among men with primary idiopathic epididymis obstruction. Excluding criteria for OA cases were azoospermia due vas deferens or ejaculatory duct. Moreover, patients with genital infections, vasectomy, or other iatrogenic injuries to the male reproductive tract were excluded from the study.
Wu X	2015	Asian	HP	Sequence	rs6165 rs6166	164/212	AA:80/95, AG:75/95, GG:9/22; AA:82/100, AG:72/92, GG:10/20;	0.108 0.261	The patients selected consisted of infertile men with idiopathic infertility ranging from oligospermia to azoospermia. Other diseases that could cause secondary infertility, such as obstructive azoospermia, karyotype abnormalities, Y chromosome microdeletions, and cryptorchidism, were excluded. The controls consisted of normospermic patients who were from couples suffering infertility due to the woman’s issues and no genetic or reproductive tract disease.
Grigorova M	2014	Caucasian	PB	Sequence	rs1394205	982/641	GG:552/380,AG:362/228,AA:68/33	0.412	The inclusion criterion for male partners of infertile couples entering the study was sperm concentration below 20 × 10^6^/ml. All men with causal factors for male factor infertility (obstruction, cryptorchidism, chromosomal abnormalities, Y chromosome deletions, hypogonadotrophic hypogonadisn, testicular diseases, sexual dysfunctions, androgen abuse, severe traumas and operation in genital area, chemo- and radio- therapy) were excluded.
Grigorova M	2013	Caucasian	HP	PCR-RELP	rs6166	1052/738	AA:379/261,AG:506/353,GG:167/124;	0.930	The inclusion criterion for male partners of infertile couples entering the study was sperm concentration below 20 × 10^6^/ml. All men with causal factors for male factor infertility (obstruction, cryptorchidism, chromosomal abnormalities, Y chromosome deletions, hypogonadotrophic hypogonadisn, testicular diseases, sexual dysfunctions, androgen abuse, severe traumas and operation in genital area, chemo- and radio- therapy) were excluded.
Lazaros L	2013	Caucasian	HP	PCR-RELP	rs6165 rs6166	250/200	AA:65/49, AG:132/92, GG:53/59; AA:65/49, AG:132/92, GG:53/59;	0.356 0.356	The study population was consisted of 450 Greek men, 250 normozoospermic and 200 oligozoospermic men, who were referred to the in vitro fertilization (IVF) Unit, which was based on World Health Organization criteria (WHO, 1999).
Song D	2013	Asian	HP	Sequence	rs6165 rs6166	200/150	AA:81/65, AG:88/63, GG:31/22; AA: 86/69, AG:87/58, GG:27/23;	0.386 0.506	Inclusion criteria were as follows: (I) diagnosed as idiopathic infertility or severe oligoasthenozoospermic (ii) sperm count below 10 × 10^6^/ml, sperm motility (a + b) = 1.19%–9.99% and normal sperm morphology >4% as determined by at least three semen analyses. Patients were excluded if they had: (I) hypogonadotrophic hypogonadism or abuse of androgenic (anabolic) steroids (ii) obstructive azoospermia (iii) underwent treatment with chemotherapeutic agents or radiotherapy. Azoospermic and severely oligozoospermic men with karyotype abnormalities and Y chromosome long arm microdeletions were excluded.
Ghirelli-Filho M	2012	Brazilian	HP	Taqman	rs6165 rs6166	217/138	AA:74/33, AG:89/72, GG:54/33; AA: 49/32, AG:88/66, GG:80/40;	0.011 0.011	Infertile men with severe oligozoospermia (SO) and non-obstructive azoospermia (NOA), with at least 1 year of infertility were included in this study. Individuals with known causes of infertility including genetic factors (chromosome anomalies, AZF [azoospermia factor] microdeletions), clinical factors (varicocele, cryptorchidism) and men whose partner had factors involved in infertility were excluded from this study. To compose the control group, 217 fertile men, who have at least 2 children by direct survey and who lacked any history of requiring assisted reproduction technology, were selected.
Li Y	2011	Asian	HP	PCR-RELP	rs6165 rs6166 rs1394205	469/176	AA:189/75,AG:230/88,GG:50/13; AA:203/80,AG:220/82,GG:46/14; GG:118/101,AG:250/96,AA:101/38	0.103 0.221 0.144	Those with a history of orchitis, cryptorchidism, varicocoele, obstruction of vas deferens, karyotype abnormality, and Y chromosome microdeletions were excluded. Additionally, subjects having special occupational exposure which may be suspected to affect semen quality (such as pesticides or other agents) were precluded. Then 364 idiopathic infertile patients were divided into three groups: 97 males with non-obstructive azoospermia, 79 with oligozoospemia (sperm count < 40 × 10^6^/ejaculum), 188 with normozoospermia (sperm count ≥ 40 × 10^6^/ ejaculum). The control group consisted of 285 subjects with normal semen parameters, all of which had fathered at least one child without assisted reproductive technologies.
Safarinejad MR	2011	Caucasian	HP	PCR-RELP	rs6165 rs6166	172/172	AA:78/62, AG:74/90, GG:20/20; AA: 85/69, AG:72/80, GG:15/23;	0.702 0.964	All the infertile patients had to have a history of primary infertility for at least 24 months with no known etiology for their infertility. A history of the following: cryptorchidism, varicocele, testicular torsion or genital surgery; azoospermia; UTIs; any endocrinopathy; Y chromosome microdeletions or karyotype abnormalities; use of cytotoxic drugs, immunosuppressants, anticonvulsives, androgens or antiandrogens; leukocytospermia (more than 106 white blood cells per mL), or a positive mixed agglutination reaction test were exclusion criteria. Participants with a history of hepatobiliary disease, significant renal insufficiency, drug or alcohol abuse or dependence, tobacco use, occupational and environmental exposures to potential reproductive toxins, and a body mass index (BMI) of ≥30 kg/m^2^ were also excluded.
Balkan M	2010	Caucasian	HP	PCR-RELP	rs6166 rs1394205	240/270	AA:154/176,AG:49/59,GG:37/35; GG:178/203,AG:49/53, AA:13/14	0.000 0.001	The study population consisted of 240 proven fathers (sperm count > 20 × 10^6^/ml and serum FSH levels < 7 IU/L), and infertile men (150 non-obstructive azoospermic and 120 severe oligozoospemic in which sperm count < 10 × 10^6^/ml). And karyotype abnormalities and Y chromosome microdeletions were exclusion criteria.
Lend AK	2010	Caucasian	HP	PCR-RELP	rs6165 rs6166 rs1394205	208/150	AA:67/50, AG:106/72, GG:35/28; AA:66/50, AG:107/73, GG:35/27; GG: 110/78, AG:85/61, AA:13/11	0.526 0451 0.522	Patients with non-obstructive idiopathic azoospermia (*n* = 36) or oligozoospermia (sperm count <20 × 10^6^/ml, *n* = 114) and without any obvious cause of infertility were considered as infertile cases for the study. The controls group consisted of 208 military conscripts selected based on their sperm count of ≥75 × 10^6^/ml.
Shimoda C	2009	Asian	HP	PCR-RELP	rs6165 rs6166	146/343	AA:68/118,AG:61/179,GG:17/46; AA:72/131,AG:62/164,GG:12/45;	0.560 0.791	All patients presented with non-obstructive azoospermia and elevated basal FSH concentrations (>10.0 mIU/mL), without Y chromosome microdeletion and normal karyotypes. Fertility status was proven by the fact that each of the control subjects had fathered one or more children.
Zalata AA	2008	Caucasian	HP	PCR-RELP	rs6166	30/52	AA: 14/18, AG:10/20, GG:6/14;	0.122	The 82 Caucasian men were divided into group (gp)1 (*n* = 30) normozoospermic, fertile and healthy volunteers who had achieved conception with 1 year, and gp2 (*n* = 52) infertile oligoasthenozoospermic males. Exclusion criteria were varicocele, cryptorchidism, karyotype anomalies, Y chromosome microdeletions and leucocytospermia.
Pengo M	2006	Caucasian	HP	PCR-RELP	rs6165 rs6166 rs1394205	351/215	AA:114/75,AG:153/96,GG:84/44; AA:114/75,AG:153/96,GG:84/44; GG:203/126,AG:121/73,AA:27/16	0.022 0.023 0.139	Inclusion criteria were as follows: (i) a minimum of 1 year of infertility (ii) sperm count below 20 × 106/ml as determined by at least two semen analyses. Patients were excluded if they had: (i) hypogonadotrophic hypogonadism or abuse of androgenic (anabolic) steroids (ii) obstructive azoospermia (iii) undergone treatment with chemotherapeutic agents or radiotherapy. Azoospermic and severely oligozoospermic men with karyotype abnormalities and Y chromosome long arm microdeletions were excluded.
Galan JJ	2005	Caucasian	PB	PCR-RELP	rs6166	95/104	AA:26/38, AG:51/49, GG:18/17;	0.428	104 Caucasoid men with idiopathic non-obstructive oligozoospermia or azoospermia (sperm counts < 5 × 10^6^/ml) were recruited during this work, which did not consider Y chromosome microdeletions as a confounding factor because of the low frequency of Y chromosome microdeletions.
Ahda Y	2005	Caucasian	PB	Taqman	rs6165 rs6166 rs1394205	186/341	AA:74/101,AG:77/166,GG:35/74; AA:74/101,AG:77/166,GG:35/74; GG:102/164,AG:74/150,AA:10/27	0.068 0.068 0.466	The study population consisted of 186 men with normal semen values according to WHO criteria (1999) and normal serum FSH levels (<7 IU/L), recruited for contraceptve trials through advertisement in the local newspaper, and 341 infertile men with nonobstructive azoospermia and elevated FSH levels (≥7 IU/L) attending our infertility clinic. Hypogonadotropic hypogonadism and genetic defects causing azoospemia (Klinefelter syndrome or deletions of the Y chromosome) were exclusion criteria.
This study	2016	Asian	HP	Mass-array	rs6165 rs6166 rs1394205	340/255	AA:163/210,AG:144/199,GG:33/52; AA:169/230,AG:146/187,GG:25/44; GG:78/112,AG:170/233,AA:92/116	0.884 0.391 0.975	The study consisted of 255 infertile men, including 166 azoospermic or severe oligozoospermia (sperm concentration < 5 ×10^6^/ml), and 89 oligozoospermia (sperm concentration5–15×10^6^/ml), with at least 1 year of infertility. Individuals with known causes of infertility including genetic factors (chromosome anomalies), AZF microdeletions, clinical factors (varicocele, crytorchidism) and infections were excluded from this study.

*HP* hospital population, *PB* population based *HWE* Hardy-Weinberg equilibrium

**Fig. 2:**
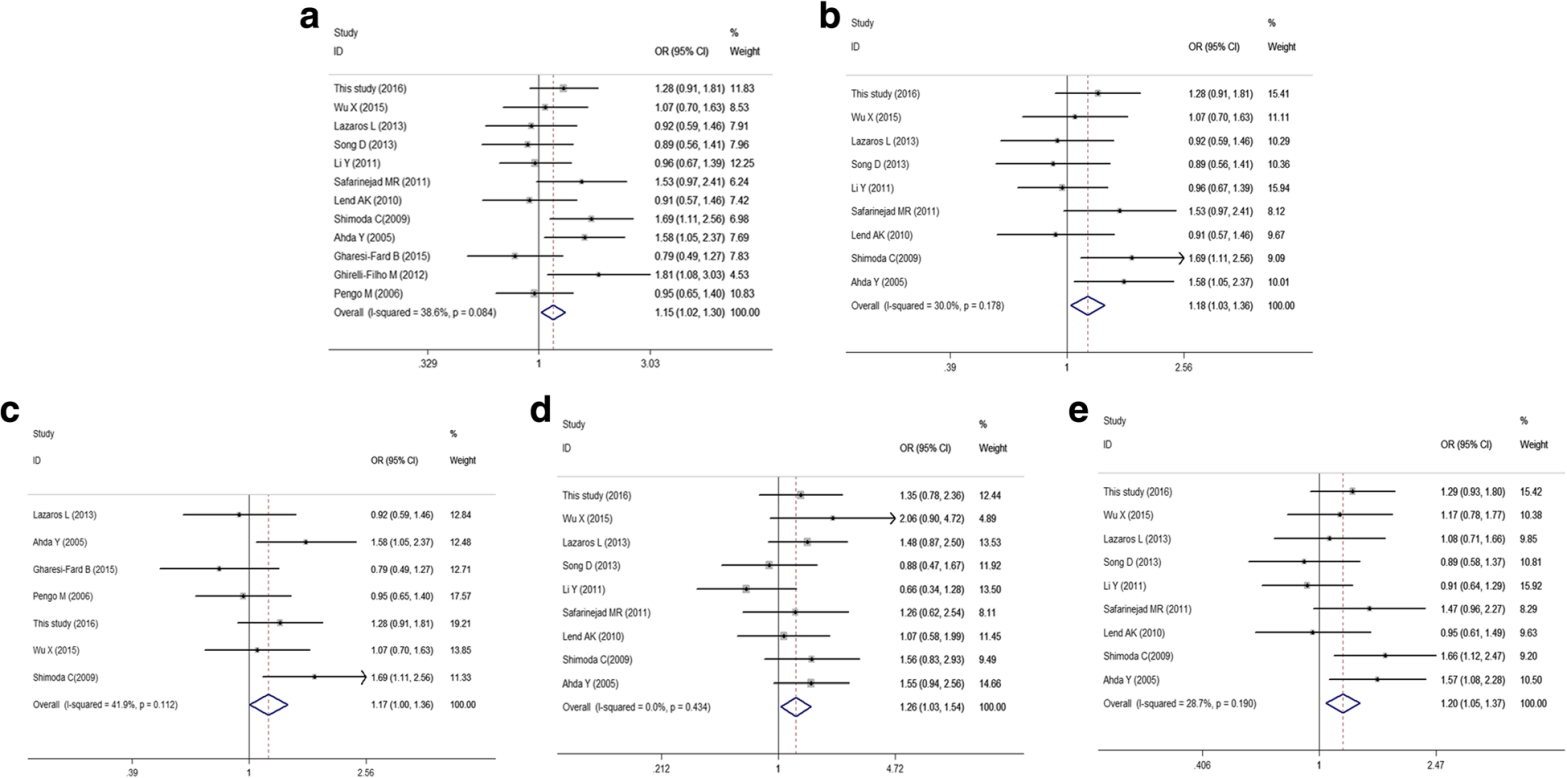
Forest plot for the association between *FSHR* rs6165 and male infertility for fixed effects model. **a** AG vs. AA in overall analysis; (**b**) AG vs. AA in subgroup analysis of HWE > 0.05; (**c**) AG vs. AA in subgroup analysis of case count > 200; (**d**) GG vs. AA in subgroup analysis of HWE > 0.05; (**e**) GG + AG vs. AA in subgroup analysis of HWE > 0.05. For each study, the point estimate of OR (the size of the square is proportional to the weight of each study) and 95% CI for OR (extending lines) is shown. Pool OR and 95%CI are presented as diamonds

For rs6165 polymorphism, the overall analyses showed that AG genotype was associated with increased risk of male infertility (OR: 1.15, 95%CI: 1.02–1.30, *P*: 0.021). In addition, in the subgroup analysis with HWE > 0.05, significant differences was observed for the genotype GG (GG vs. AA, OR:1.26, 95%CI:1.03–1.54, *P*:0.023), AG (AG vs. AA, OR:1.18, 95%CI:1.03–1.36, *P*:0.018) and GA + GG (GA + GG vs. AA, OR:1.20, 95%CI:1.05–1.37, *P*: 0.006). By the race, one paper which reported in the Brazilian population, revealed a significant association for the comparison of AG vs. AA (OR: 1.81, 95%CI: 1.08–3.31, *P*: 0.023), GA + GG vs. AA (OR: 1.65, 95%CI: 1.02–2.67, *P*: 0.042). And a slight significant association for the AG vs. AA (OR: 1.17, 95%CI: 1.00–1.36, *P*: 0.047) was found in the subgroup analysis of case count > 200. However, no significant association was revealed in the remaining subgroup analyses, as summarized in Table 5, Fig. 2.

**Table 5:** Stratification analyses of genetic susceptibility of *FSHR* rs6165, rs6166, rs1394205 polymorphisms to male infertility

Variables	Cases/controls	GG vs. AA				AG vs. AA				AG + GG vs. AA				GG vs. AA + AG			
Rs6165		OR (95% CI)	*P*	*P*_*h*_	*I* ^*2*^	OR (95% CI)	*P*	*P*_*h*_	*I* ^*2*^	OR (95% CI)	*P*	*P*_*h*_	*I* ^*2*^	OR (95% CI)	*P*	*P*_*h*_	*I* ^*2*^
Total	2564/2903	1.11 (0.94–1.31)	0.218	0.107	35.4%	**1.15 (1.02–1.30)**	**0.021**	0.084	38.6%	1.15 (0.98–1.34) ^b^	0.092	0.042	45.6%	1.03 (0.89–1.20)	0.685	0.277	17.0%
HWE																	
> 0.05	1999/2135	**1.26 (1.03–1.54)**	**0.023**	0.434	0	**1.18 (1.03–1.36)**	**0.018**	0.178	30.0%	**1.20 (1.05–1.37)**	**0.006**	0.190	28.7%	1.17 (0.98–1.41)	0.091	0.522	0
Race																	
Caucasian	1290/1367	1.05 (0.84–1.30)	0.684	0.085	48.3%	1.09 (0.91–1.30)	0.342	0.122	42.5%	1.08 (0.92–1.28)	0.358	0.059	53.0%	1.02 (0.85–1.24)	0.820	0.146	39.0%
Asian	1136/1319	1.17 (0.88–1.55)	0.279	0.166	38.3%	1.16 (0.97–1.38)	0.107	0.209	31.8%	1.16 (0.98–1.37)	0.086	0.139	42.5%	1.08 (0.82–1.41)	0.597	0.302	17.6%
Brazilian	138/217	1.37 (0.76–2.49)	0.301	-		**1.81 (1.08–3.31)**	**0.023**	-		**1.65 (1.02–2.67)**	**0.042**	-		0.95 (0.58–1.56)	0.836	-	
Case count																	
> 200	1778/1637	1.20 (0.88–1.65) ^b^	0.254	0.038	55.1%	**1.17 (1.00–1.36)**	**0.047**	0.112	41.9%	1.17 (0.94–1.45) ^b^	0.163	0.044	53.7%	1.08 (0.91–1.30)	0.380	0.092	44.8%
< 200	786/1266	1.02 (0.77–1.35)	0.913	0.525	0	1.13 (0.93–1.38)	0.222	0.111	46.8%	1.10 (0.91–1.32)	0.329	0.140	42.2%	0.93 (0.72–1.21)	0.591	0.810	0
Rs6166		GG vs. AA				AG vs. AA				AG + GG vs. AA				GG vs. AA + AG			
Total	3728/4320	1.12 (0.98–1.29)	0.099	0.263	16.6%	1.07 (0.97–1.18)	0.205	0.514	0	1.08 (0.98–1.19)	0.121	0.300	13.4%	1.07 (0.94–1.20)	0.306	0.325	11.2%
HWE																	
> 0.05	2893/3312	**1.24 (1.05–1.45)**	**0.009**	0.395	5.1%	1.06 (0.95–1.19)	0.307	0.331	11.7%	1.10 (0.99–1.22)	0.077	0.192	25.6%	**1.20 (1.04–1.39)**	**0.013**	0.738	0
Race																	
Caucasian	2454/2784	1.11 (0.94–1.30)	0.218	0.307	14.7%	1.07 (0.95–1.22)	0.267	0.328	12.5%	1.08 (0.96–1.21)	0.204	0.208	25.6%	1.06 (0.92–1.23)	0.403	0.456	0
Asian	1136/1319	1.29 (0.97–1.73)	0.085	0.292	19.2%	1.05 (0.88–1.25)	0.614	0.442	0	1.09 (0.92–1.29)	0.324	0.284	20.5%	1.27 (0.96–1.68)	0.099	0.479	0
Brazilian	138/217	0.77 (0.43–1.38)	0.371	-		1.15 (0.66–1.99)	0.621	-		0.97 (0.58–1.61)	0.894	-		0.70 (0.44–1.12)	0.127	-	
Case count																	
> 200	2786/2929	**1.18 (1.00–1.38)**	**0.047**	0.241	22.7%	1.10 (0.98–1.24)	0.111	0.452	0	1.11 (0.99–1.24)	0.054	0.319	13.8%	1.11 (0.96–1.28)	0.168	0.242	22.6%
< 200	942/1391	0.99 (0.77–1.29)	0.931	0.376	6.7%	0.99 (0.82–1.19)	0.888	0.495	0	0.99 (0.83–1.18)	0.891	0.348	10.7%	0.97 (0.77–1.22)	0.777	0.459	0
Rs1394205		AA vs. GG				AG vs. GG				AA + AG vs. GG				AA vs. GG + AG			
Total	2048/2776	0.90 (0.63–1.28) ^b^	0.541	0.022	59.4%	0.91 (0.71–1.15)^b^	0.422	0.004	68.9%	0.91 (0.71–1. 17)^b^	0.468	0.001	74.4%	0.93 (0.77–1.12)	0.440	0.394	4.3%
HWE																	
> 0.05	1563/2185	0.89 (0.56–1.44) ^b^	0.643	0.006	72.3%	0.89 (0.64–1.24)^b^	0.489	0.001	68.9%	0.90 (0.63–1.27) ^b^	0.543	0.000	82.6%	0.92 (0.75–1.14)	0.440	0.181	36.0%
Race																	
Caucasian	1617/1967	0.93 (0.70–1.23)	0.612	0.383	4.1%	0.98 (0.85–1.14)	0.832	0.698	0	0.97 (0.85–1.12)	0.708	0.453	0	0.93 (0.71–1.23)	0.622	0.524	0
Asian	431/809	0.73 (0.27–1.98)^b^	0.537	0.002	89.3%	0.70 (0.29–1.66)^b^	0.412	0.001	90.1%	0.71 (0.28–1.77)^b^	0.458	0.000	92.1%	0.92 (0.71–1.21)	0.550	0.080	67.4%
Case count																	
> 200	1722/2099	0.99 (0.77–1.26)	0.903	0.294	19.0%	0.99 (0.86–1.15)	0.930	0.661	0	0.99 (0.86–1.13)	0.836	0.388	3.2%	0.99 (0.79–1.25)	0.958	0.430	0
< 200	326/677	0.68 (0.26–1.79) ^b^	0.430	0.043	75.5%	0.67 (0.30–1.48)^b^	0.319	0.005	87.5%	0.67 (0.30–1.54) ^b^	0.347	0.002	89.5%	0.78 (0.54–1.12)	0.176	0.268	18.4%

^a^ *P* value of Q-test heterogeneity test

^b^Random-effect model was used when a P_H_ < 0.05, otherwise, fixed-effects model was used; Bold numbers mean statistically significant results

*OR* odds ratio, *CI* confidence interval, *P*_*H*_ *P* value of heterogeneity, *I* ^*2*^ *:0–25* no heterogeneity, *20–50* modest

**Fig. 3:**
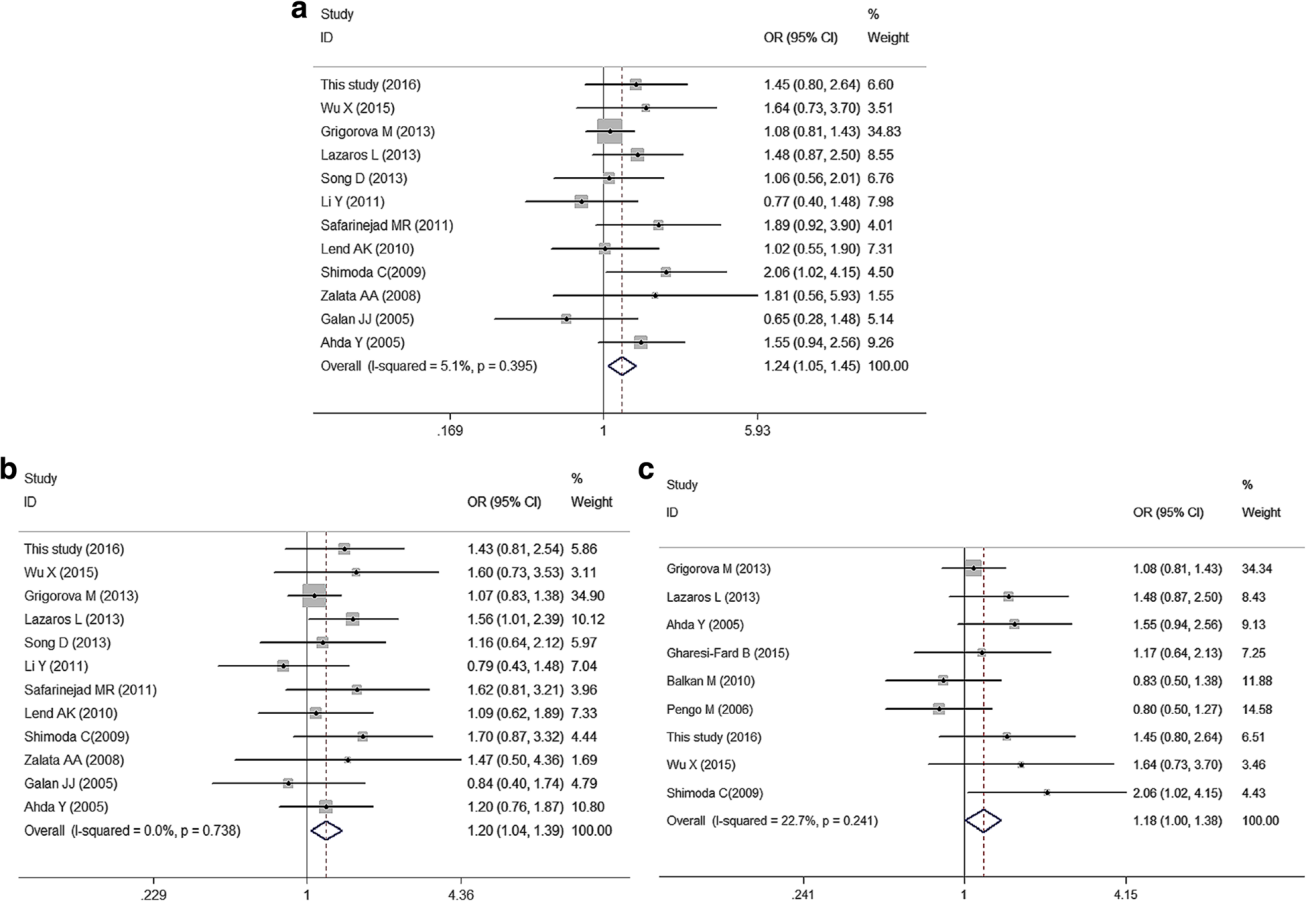
Meta-analysis of male infertility risk associated with *FSHR* rs6166 for fixed effects model. **a** GG vs. AA in subgroup analysis of HWE > 0.05; (**b**) GG vs. AA + AG in subgroup analysis of HWE > 0.05; (**c**) GG vs. AA in subgroup analysis of case count > 200

For rs6166 polymorphism, no significant association was observed under the all genetic models in overall analyses; however, significant differences was showed for the comparison of GG vs. AA (OR:1.24, 95%CI:1.05–1.45, *P*: 0.009), GG vs. AA + AG (OR:1.20, 95%CI:1.04–1.39, *P*:0.013) in subgroup analysis with HWE > 0.05, and the comparison of GG vs. AA (OR:1.18, 95%CI:1.00–1.38, *P*:0.047) in subgroup analysis with case count >200, as presented in Table 5, Fig. 3.

For rs1394205 polymorphism, no significant association was showed in overall analyses and subgroup analyses.

### Test of heterogeneity and sensitivity analysis

Sensitivity analyses on the *FSHR* SNPs rs6165, rs6166 and rs1394205 under four models were conducted. Among rs6165 polymorphism, we found slight heterogeneity for the comparison of dominant model (GA + GG vs. AA: *P*_*h*_: 0.042) in overall analysis, homozygote model (GG vs. AA: *P*_*h*_: 0.038), dominant model (GA + GG vs. AA: *P*_*h*_: 0.044) in subgroup analysis of case count > 200. The heterogeneity was decreased respectively to 0.120, 0.266 and 0.201 when omitting the paper reported by Gharesi-Fard et al. [13]. Among rs1394205 polymorphisms, a significant heterogeneity was apparent in the overall analyses under the homozygote model, heterozygous model and dominant model. Consistently, subgroup analyses by HWE, race and case count for rs1394205 also showed a significant heterogeneity, which indicated in Table 5. Sensitivity analysis revealed that the study reported by Li Y et al. [28], was the origin of substantial heterogeneity, and this was decreased when it was removed.

### Publication bias

Begg’s Funnel plots and Egger’ test were performed to assess publication bias. For the *FSHR* rs6165, rs6166 and rs1394205 funnel plot shape did not show any evidence of obvious asymmetry in all comparison models. And the Egger’s test was used to provide statistical evidence for funnel plot symmetry, which showed no obvious evidence of publication bias.

## Discussion

In the present study, we included 340 fertile males and 255 infertile males, consisting of 166 with azoospermia or severe oligozoospermia, and 89 with oligospermia, and we investigated the association between *FSHB*, *FSHR* gene polymorphisms and male infertility. Compared with the fertile controls, the infertile patients had higher FSH and LH levels, and lower sperm concentration and sperm motility. Then the most common *FSHR* allelic variants in the core promoter and exon 10, and the *FSHB* variants in the core promoter were genotyped with respect to male infertility status using the Mass ARRAY platform. However, the distributions of *FSHB* and *FSHR* allele, genotype frequencies among azoospermic, severe oligozoospermic, or oligozoospermic men and fertile men based on age-adjusted estimates were similar. However a more precise analysis should be conducted if all individual raw data were available, to allow for the adjustment according to hormone, sperm parameters and other lifestyle factors. Consistent with previous reportes [31, 34], we also identified a significant association for the comparison of GAA (*P*: 0.022, OR: 0.63, 95%CI: 0.43–0.94) among the oligozoospermic men in haplotype analysis, which indicated that the GAA haplotype would exert protective effects against male sterility.

Previous studies examining potential associations between *FSHR* polymorphisms and male infertility parameters have produced contradictory results. Variants of *FSHR* have been shown to affect the serum FSH, inhibin B, anti-Mullerian hormone (AMH) and total testes volume [5, 14, 25, 33]. However, the majority of studies have failed to detect any link between common *FSHR* isoforms and male reproductive parameters. It is regrettable that not all clinical fertility parameters in this study were collected, as we could not find any association between the clinical fertility parameters and the *FSHB* and *FSHR* genotypes.

FSH secreted by the anterior pituitary, together with other endocrine factors, plays a central role in establishing and maintaining human fertility. In males, circulating FSH stimulates gametogenesis and steroidogenesis in the gonads by binding to its receptor (FSHR). Tuttelmann et al. reported that the *FSHB* -211G > T T-allele showed significant dosage effects on FSH, LH and bilateral testicular volume. Moreover, *FSHR* 2029A > G significantly modulated the more dominant effect of *FSHB* -211G > T on serum FSH and testicular volume among the T-allele carriers [18], suggesting that the interplay between polymorphisms in hormone and receptor is of relevance under physiological conditions.

We searched available databases, such as GWAS Central, National Human Genome Research Institute GWAS Catalog and PUBMED, but failed to find a relevant genome-wide association study (GWAS) on all SNPs. To date, no clear consensus appears to have been reached in the literature on the relationship between *FSHR* polymorphisms and male infertility risk.

To resolve the conflicting results, we carried out a meta-analysis to obtain a more precise estimation of the associations. Contrary to previous meta-analyses that found no significant association between *FSHR* rs1394205 (G-29A), rs6165 (Thr307Ala), or rs6166 (Asn680Ser) polymorphisms and the risk of male infertility [2, 30, 34], this study indicated that the rs6165G allele was associated with increased risk of male infertility, particularly in the subgroup analysis of HWE > 0.05. In addition the rs6166 GG genotype was also observed to be a risk factor for infertility in the subgroup analysis of HWE > 0.05. One possible reason for these discrepant findings is specific selection of the publications. However, the *FSHR* rs1394205 polymorphism was not associated with male infertility, which was similar to the findings of other studies.

In addition, identifying the source of heterogeneity is one of the most important goals of the meta-analysis. In each case, the heterogeneity could be a result of differences in ethnicity, sources of controls, methods used and so on. In this study, we found that significant heterogeneity existed in overall analysis and sub-group analysis for the comparison of *FSHR* rs1394205 polymorphisms and male infertility. Sensitivity analysis revealed that the study reported by Li et al. [28], contained substantial heterogeneity, and this was decreased when it was removed. The subjects of the case-control study by Li et al. [28], comprised 364 idiopathic infertile patients (97 with non-obstructive azoospermia, 79 with oligozoospermia and 188 with normozoospermia) and 285 fertile men were collected [28]. However, previously- reported meta-analyses including our meta-analysis considered 188 men with normozoospermia and 285 fertile men as controls, which brought out different associations between the *FSHR* rs1394205A allele and male infertility, resulting in the significant heterogeneity.

It should be pointed out that there are some limitations in this meta-analysis. Firstly, inadequate sample size and inappropriate control subjects resulted in limited power for exploring the real association, and subgroup analyses by HWE, ethnicity, and case count involved relatively small groups, which were more likely to reveal greater beneficial effects than a large-scale trial. Secondly, owing to lack of the original data, we could not further evaluate the potential interactions between genes, which might affect male infertility. Thirdly, much detailed information, including body mass index, age, work, smoking or alcohol habits, environmental exposure and other lifestyle factors, is not available, so that our results were based on unadjusted estimates. A more precise analysis should be conducted through adjustment by other co-variants. In fact, some other genes as well as environmental exposure could also play an important role in spermatogenesis.

Owing to the critical role of FSH in spermatogenesis, polymorphisms in the *FSHB* and *FSHR* genes might disturb normal spermatogenesis and affect male reproductive ability. Because of this, FSH treatment has always been tempting and is actively prescribed by many doctors even though the efficacy of FSH therapy remains a contentious issue. One study showed that patients with at least one *FSHR* c.2039A > G G allele had a significant increase in total sperm count after 3 months of treatment with recombinant FSH (rFSH, 150 IU/three times per week). Another study considered the *FSHB*-211G > T genotype and showed that TT homozygote, representing 25% of men with oligozoospermia and low FSH levels, could significantly benefit from FSH treatment [23]. However, there have been other studies showing that the outcome of FSH treatment was poor. Possible reasons include that the doses used were too low, with a high dose of recombinant rFSH necessary for treatment to be effective. Another explanation is that FSH therapy might require stratification of men according to genotype into FSH-responsive and non-responsive individuals, which depending on the *FSHB* and *FSHR* genotypes [36].

## Conclusions

This study suggested that *FSHR* GAA haplotype would exert protective effects against male sterility, which indicated that the combination of three SNP genotypes of *FSHR* was predicted to have a much stronger impact than either one alone. Then in the meta-analysis, a significant association was seen between *FSHR* rs6165, rs6166 polymorphisms and male infertility. In terms of male infertility with multifactorial etiology, further studies with larger sample sizes and different ethnic backgrounds or other risk factors are warranted to clarify the potential role of *FSHB* and *FSHR* polymorphisms in the pathogenesis of male infertility.

## Abbreviations

AMH: Anti-Mullerian hormone
AR: Androgen receptor
CI: Confidence interval
CNKI: Chinese national knowledge infrastructure
DAZL: Deleted in azoospermia like gene
DSD: Disorders of sexual development
E: Estradiol
FSHR: Follicle stimulating hormone receptor gene
FSHβ: FSH beta subunit
GC: Granulosa cells
GWAS: Genome-wide association study
hGLC: Human granulosa-lutein cells
HWE: Hardy-Weinberg equilibrium
KS: Klinefelter syndrome
LH: Luteinizing hormone
MTHFR: Methylene- tetrahydrofolate reductive
OR: Odds ratio
SNP: Single nucleotide polymorphism
T: Testosterone
vs.: Versus
